# Low-Load High Volume Resistance Exercise Stimulates Muscle Protein Synthesis More Than High-Load Low Volume Resistance Exercise in Young Men

**DOI:** 10.1371/journal.pone.0012033

**Published:** 2010-08-09

**Authors:** Nicholas A. Burd, Daniel W. D. West, Aaron W. Staples, Philip J. Atherton, Jeff M. Baker, Daniel R. Moore, Andrew M. Holwerda, Gianni Parise, Michael J. Rennie, Steven K. Baker, Stuart M. Phillips

**Affiliations:** 1 Exercise Metabolism Research Group, Department of Kinesiology, McMaster University, Hamilton, Ontario, Canada; 2 School of Graduate Entry Medicine and Health, City Hospital, University of Nottingham, Derby, United Kingdom; 3 Department of Medical Physics and Applied Radiation Sciences, McMaster University, Hamilton, Ontario, Canada; 4 Department of Neurology, Michael G. DeGroote School of Medicine, McMaster University, Hamilton, Ontario, Canada; Universidad Europea de Madrid, Spain

## Abstract

**Background:**

We aimed to determine the effect of resistance exercise intensity (% 1 repetition maximum—1RM) and volume on muscle protein synthesis, anabolic signaling, and myogenic gene expression.

**Methodology/Principal Findings:**

Fifteen men (21±1 years; BMI = 24.1±0.8 kg/m^2^) performed 4 sets of unilateral leg extension exercise at different exercise loads and/or volumes: 90% of repetition maximum (1RM) until volitional failure (90FAIL), 30% 1RM work-matched to 90%FAIL (30WM), or 30% 1RM performed until volitional failure (30FAIL). Infusion of [*ring*-^13^C_6_] phenylalanine with biopsies was used to measure rates of mixed (MIX), myofibrillar (MYO), and sarcoplasmic (SARC) protein synthesis at rest, and 4 h and 24 h after exercise. Exercise at 30WM induced a significant increase above rest in MIX (121%) and MYO (87%) protein synthesis at 4 h post-exercise and but at 24 h in the MIX only. The increase in the rate of protein synthesis in MIX and MYO at 4 h post-exercise with 90FAIL and 30FAIL was greater than 30WM, with no difference between these conditions; however, MYO remained elevated (199%) above rest at 24 h only in 30FAIL. There was a significant increase in Akt^Ser473^ at 24h in all conditions (P = 0.023) and mTOR^Ser2448^ phosphorylation at 4 h post-exercise (P = 0.025). Phosporylation of Erk1/2^Tyr202/204^, p70S6K^Thr389^, and 4E-BP1^Thr37/46^ increased significantly (P<0.05) only in the 30FAIL condition at 4 h post-exercise, whereas, 4E-BP1^Thr37/46^ phosphorylation was greater 24 h after exercise than at rest in both 90FAIL (237%) and 30FAIL (312%) conditions. Pax7 mRNA expression increased at 24 h post-exercise (P = 0.02) regardless of condition. The mRNA expression of MyoD and myogenin were consistently elevated in the 30FAIL condition.

**Conclusions/Significance:**

These results suggest that low-load high volume resistance exercise is more effective in inducing acute muscle anabolism than high-load low volume or work matched resistance exercise modes.

## Introduction

Resistance exercise stimulates the synthesis of skeletal muscle proteins [1], [2], which is eventually expressed as muscle hypertrophy [3], [4]. It is commonly recommended that high-load contractions (i.e., ≥70% of 1 repetition maximum; 1RM) be performed to provide an optimal stimulus for muscle growth [5]. It has recently been established, however, that myofibrillar (MYO) protein synthesis is already maximally stimulated at 60% 1RM, in the post-absorptive state, with no further increase at higher load intensities (i.e., 75–90% 1RM) [6]. Additionally, performance of low-load contractions (∼20% 1RM) with vascular occlusion is sufficient to induce an increase in mixed muscle (MIX) protein synthesis [7], which explains the significant improvements in muscle size and strength, equivalent to those seen at higher contractile intensities, that occur with blood flow occluded training [8], [9], [10]. Collectively, these data suggest that heavy (i.e., high intensity) external loads are not a prerequisite to elicit increases in muscle protein synthesis [7] and ultimately muscle hypertrophy [8], [9], [10].

Henneman's work [11] described the recruitment of motor units as occurring in a progressive fashion from small to large (i.e., the size principle). As opposed to the requirement for high intensity contractions we posited that the total volume of contractions, independent of intensity, would result in full motor unit activation and muscle fibre recruitment and would be of equal or greater importance as intensity to the acute stimulation of muscle protein synthesis. Specifically, the same degree of muscle fibre activation and presumably a similar stimulation of myofibrillar (MYO) protein synthesis, would occur regardless of intensity provided that the exercise was performed until volitional fatigue (failure) in line with observations from occlusion training [8], [9], [10].

The regulation of muscle protein synthesis is multifaceted and recent investigations have demonstrated such signalling pathways as Akt-mTOR and mitogen-activated protein kinase (MAPKs; e.g., Erk1/2) cascades as important promoters of exercise-induced anabolism [6], [12], [13], [14], [15], [16]. However, from these investigations it is difficult to discern if the exercise anabolic signalling proteins are activated for longer periods (e.g., ≥24 h) [17] and playing an important role in sustaining increases in muscle protein synthesis shown to occur in the days after exercise [18], [19]. Similarly, adult muscle progenitor (satellite) cells have been suggested to be essential for muscle hypertrophy as an adaptation to resistance exercise [20]. However, it is difficult to ascertain the importance of increased expression of Pax7 [21], a marker of satellite cell activation, which together with other myogenic regulatory factors (MRFs), such as MyoD, Myf5, MRF4, and myogenin, which are involved in the activation, proliferation, and terminal differentiation of muscle stem cells [21], [22], are related to the acute exercise induced muscle protein synthetic response, especially at later time points (e.g., >24 h) beyond the exercise bout.

In the present study, we aimed to systematically investigate the impact of two distinctly different exercise loads along with differing exercise volumes on anabolic signaling, myogenic gene expression, and rates of muscle protein synthesis (MIX, MYO, SARC). Specifically, we utilized a unilateral model in which subjects performed exercise at 90% 1RM until failure (90FAIL), 30% 1RM in which the amount of external work was matched to 90FAIL (30WM), or 30% 1RM to failure (30FAIL). This provided us with a tool with which to unravel the separate influences of load (intensity) and volume on specific anabolic variables after acute resistance exercise. We hypothesized that the anabolic response to exercise would be similar between exercise bouts designed to elicit maximal fibre activation (i.e., 90FAIL and 30FAIL) [11]; however, exercise intensity would important to maximize the anabolic response between work-match exercise modes (90FAIL>30WM) [6].

## Methods

### Subjects

Fifteen healthy recreationally active men (21±1 years; BMI = 24.1±0.8 kg/m^2^, means±S.E.M.) volunteered to participate in the study. Participants reported engaging in lower body exercise such as resistance exercise alone or in combination with cycling more than 3 times weekly for the prior 6 months.

### Ethics Statement

All participants were informed of the purpose of the study, the experimental procedures involved and all the potential risks involved before obtaining written consent. All participants were deemed healthy based on their response to a routine medical screening questionnaire. The study was approved by the local Health Sciences Research Ethics Board of McMaster University and conformed to standards for the use of human subjects in research as outlined in the current *Declaration of Helsinki* (http://www.wma.net/en/30publications/10policies/b3/index.html).

### Experimental design

To minimize neuromuscular-based gains in strength and to increase the reliability of the strength measurement we chose to study individuals who were familiar with lower body exercise. At least two weeks before the infusion protocols, maximal strength tests were determined for each leg as a one repetition maximum (1RM; i.e., the maximal amount of weight lifted for one repetition during a concentric contraction, using a standard leg extension machine (Badger 2001 series by Magnum Fitness Systems, South Milwaukee, WI, USA). The values were confirmed on a subsequent visit to the laboratory. Only one subject needed three attempts to determine a 1RM. There was no difference (P = 0.77) between the subject's unilateral 1RM for the right (84.4±4.6 kg) and left leg (84.4±4.2 kg). After strength testing, subjects were randomized in a counterbalanced fashion for leg strength and body weight to perform unilateral resistance exercise with a load that corresponded to two of three conditions: (i) 90% 1RM (90FAIL); (ii) 30% 1RM in which the external work (repetitions×load) was work-matched to 90FAIL (30WM); and (iii) 30% 1RM in which the subjects performed the sets until volitional failure (30FAIL). A unilateral model (n = 10 per group) was chosen to reduce between-subject variability and most easily facilitate the comparison of three conditions in subjects with only two opportunities for study. We acknowledge this model did not allow for a complete within-subject design, as all research participants did not complete each prescribed exercise load, it nevertheless provided reduced variability compared to a complete between subject design. We expected this design to increase the chance of detecting any differences deriving from in muscle protein synthesis and molecular events that occurred as a result of the different loads utilized rather than those due to between-subject factors (i.e., genetics, motivation, etc.). A technical difficulty was encountered during one experimental trial which precluded our ability to determine muscle protein synthesis on this subject and therefore resulted in n = 9 for the 90FAIL and 30WM conditions.

### Dietary and activity control

Each subject was instructed to eat no later than 2200 h on the day preceding the trial. Subjects recorded their dietary intake for three days before the resting muscle protein synthesis measurement. This intake was replicated before the exercise muscle protein synthesis measurement. On the morning of the exercise trial (2 h prior to arrival to the laboratory), subjects consumed a liquid meal (Ensure plus, Abbott Laboratories, Saint-Laurent, Quebec; 61% carbohydrate, 15% protein, and 24% fat) that provided ∼15% of the estimated daily caloric need (<1890 kJ) to standardize the composition, amount, and timing of the morning breakfast before beginning the 4 h post-exercise muscle protein synthesis determination. After the 4 h post-exercise muscle protein synthesis measurement, subjects ingested no later than 2200 a similar meal to that previously logged in the three day diet diary log. This dietary approach has been used by us previously [1], [23], [24]. However, it is important to recognize that other dietary control protocols are commonly utilized to study protein metabolism that eliminate any potential for subject noncompliance [14], [25]. Subjects were asked to refrain from physical activity for three days prior to each protein synthesis study and within 24 h of the exercise bout.

### Infusion protocol

On trial days, participants reported to the laboratory and a catheter was inserted in the antecubital vein of one arm for blood sampling. After a baseline blood sample was obtained, a second catheter was inserted in the contra-lateral arm for the primed-constant infusion of L-[*ring*-^13^C_6_]phenylalanine (prime: 2 µmol kg^−1^, 0.05 µmol kg^−1^ min^−1^; Cambridge Isotope Laboratories, Woburn, MA, USA). Blood samples were drawn every hour and processed as previously described [26].

During trial 1 only one biopsy was obtained, and the baseline blood was utilized for the calculation of resting muscle protein synthesis (see calculation below [1]), whereas during trial 2 and 3 bilateral biopsies were taken to characterize the post-exercise responses. Muscle biopsies were performed with a Bergström needle that was custom-modified for manual suction under local anaesthesia (2% Xylocaine). All muscle biopsies (∼100 mg) were obtained through a separate incision from the *vastus lateralis*. Biopsy samples were blotted and freed of any visible fat and connective tissue, immediately frozen in liquid nitrogen and stored at −80°C until further analysis. The same infusion protocol was repeated on trial 1 (rest), trial 2 (4 h post-exercise), and trial 3 (24 h post-exercise). During the infusions subjects rested comfortably on a bed.

### Resistance exercise

Upon arriving at the laboratory, the load was set for each subject according to the previously established 1RM. For the 90FAIL and 30FAIL leg, participants performed the exercise and were given verbal encouragement until concentric failure. Failure was recognized when a complete range of motion for the exercise could not be completed. During the 30WM trial subjects performed the same amount of external work as in the 90FAIL trial and therefore exercise was not performed to failure. For all training loads, subjects performed four sets and were given three minutes rest between sets. The subjects were instructed on proper lifting cadence based on verbal cues and a metronome set to 50 beats per min, which corresponded to 1-s concentric muscle action, 0-s pause, and a 1-s eccentric muscle action. Time-under-tension was recording with a standard chronometer.

### Immunoblotting

Muscle biopsies (∼40–50 mg) were homogenized in ice-cold extraction buffer (7.5 µl/mg^−1^) containing 50mM Tris-HCl (pH 7.4), 1mM EDTA, 1mM EGTA, 10mM ß-glycerophosphate, 50mM NaF, 0.5mM activated sodium orthovanadate (all Sigma Aldrich, Poole, UK) and a complete protease inhibitor cocktail tablet (Roche, West Sussex, UK). Homogenates were centrifuged at 2200×g for 10 min at 4°C, before recovery of supernatants representing sarcoplasmic fractions. Approximately 200 µl aliquot was saved for determination of sarcoplasmic protein synthesis and the remainder was used for immunoblotting. Bradford assays were used to determine sarcoplasmic protein concentrations after which samples were standardized to 3 mg/ml^−1^ by dilution with 3× Laemmli loading buffer in order to measure relative phosphorylated protein concentrations of Akt^Ser473^, eEF2^Thr56^, mTOR^Ser2448^, p70S6K^Thr389^, 4EBP1^Thr37/46^, Erk1/2^Tyr202/204^, (New England Biolabs, UK) and α-actin (Sigma Aldrich, Poole, UK). Samples were mixed and heated at 95°C for 5 min before fifteen micrograms of protein/lane was loaded on to Criterion XT Bis-Tris 12% SDS-PAGE gels (Bio-Rad, Hemel Hempstead, UK) for electrophoresis at 200V for ∼60 min. Gels were equilibrated in transfer buffer (25mM Tris, 192mM glycine, 10% methanol) for 30 min before proteins were electroblotted on to 0.2 µm PVDF membranes (Bio-Rad) at 100V for 45 min. After blocking with 5% low-fat milk in TBS-T (Tris Buffered Saline and 0.1% Tween-20; both Sigma-Aldrich, Poole, UK) for 1 h, membranes were rotated overnight with primary antibody against the aforementioned targets at a concentration of 1∶2000 at 4°C. Membranes were washed (3×5 min) with TBS-T and incubated for 1 h at room temperature with HRP-conjugated anti-rabbit secondary antibody (New England Biolabs, UK), before further washing (3×5 min) with TBS-T and incubation for 5 min with ECL reagents (enhanced chemiluminescence kit, Immunstar; Bio-Rad). Blots were imaged and quantified by assessing peak density after ensuring bands were within the linear range of detection using the Chemidoc XRS system (Bio-Rad, Hemel Hempstead, UK). Phosphorylation of signalling proteins was corrected for loading anomalies to α-actin. Immunoblot data is expressed as the ratio of phosphorylated to total protein and displayed as fold changes from resting.

### RNA isolation

Approximately 20–25 mg of wet muscle from the 24 h post-exercise sampling period was utilized as we have previously established that MRFs mRNA expression is robust above rest at this time point [27]. RNA was isolated from homogenized muscle samples using TRIzol method and a Total RNA Kit as previously decribed [27]. Briefly, each muscle sample was homogenized in a total of 1.0 ml of TRIzol Reagent (Invitrogen Corporation, Canada) using a rotary homogenizer. Homogenized samples were incubated at room temperature for 5 min followed by the addition of 0.2 ml of chloroform then shaken vigorously for 15 s. After another 5 min of incubation at room temperature, samples were centrifuged at 12 000×g at 4°C for 10 min. The aqueous phase was then transferred to a new tube and the volume was measured. 1 volume of 70% ethanol was added to the aqueous phase and mixed. Multiple 700 µL aliquots were then transferred into an Omega Bio-Tek Total RNA Isolation Kit spin column and purified, following the manufacturer's instructions. The RNA was quantified and purity was assessed using a spectrophotometer (NanoDrop 1000, Thermo Scientific, West Palm Beach, FL).

### Reverse Transcription (RT)

Individual samples were reverse transcribed in 20 µL reactions using a commercially available kit (Applied Biosystems High Capacity cDNA Reverse Transcription Kit; Applied Biosystems, Carlsbad, CA, USA) according to the manufacturer's instructions. The cDNA synthesis reaction was carried out using an Eppendorf Mastercycle epgradient thermal cycler (Eppendorf, Mississauga, ON, Canada).

### Quantitative real-time polymerase chain reaction (qRT-PCR)

Individual 25 µL reactions were prepared in 0.2 mL Stratagene PCR tubes (Stratagene, La Jolla, CA) and run in duplicate for each time-point. Primers were custom-made using published sequences (see table 1) and were re-suspended in 1× TE buffer (10 mM Tris-HCl, 0.11 mM EDTA) and stored at −20°C prior to use. In each reaction tube, 1.0 µl of cDNA and 7.5 µl of ddH2O were added to 16.5 µl of a master mix containing 12.5 µl of RT2 Real-Time SYBR Green/Rox PCR master mix (SuperArray Bioscience Corp., Frederick, MD) along with 2 µl of the specific forward and reverse primers (Table 1). Reactions for qRT-PCR were carried out using a Stratagene Mx3000P real-time PCR System (Stratagene, La Jolla, CA) using Stratagene MxPro QPCR Software Version 3.00 (Stratagene, La Jolla, CA). Fold changes in gene expression were calculated using the delta-delta Ct method [28], normalized to the housekeeping gene glyceraldehyde 3-phosphate dehydrogenase (GAPDH) [27]. Thus, mRNA values were expressed as a fold change from rest.

**Table 1:** qRT-PCR Primer Sequences.

Gene Name	Forward Sequence	Reverse Sequence
*Pax7*	5′-GCTCCGGGGCAGAACTACC-3′	5′-GCACGCGGCTAATCGAACTC-3′
*Myf5*	5′-ATGGACGTGATGGATGGCTG-3′	5′-GCGGCACAAACTCGTCCCCAA-3′
*MyoD*	5′-GGTCCCTCGCGCCCAAAAGAT-3′	5′-CAGTTCTCCCGCCTCTCCTAC-3′
*Myogenin*	5′-CAGTGCACTGGAGTTCAGCG-3′	5′-TTCATCTGGGAAGGCCACAGA-3′
*MRF4*	5′-CCCCTTCAGCTACAGACCCAA-3′	5′-CCCCCTGGAATGATCGGAAAC-3′
*GAPDH*	5′-CCACCCATGGCAAATTCC-3′	5′-TGGGATTTCCATTGATGACAA-3′

### Muscle Protein Synthesis

Intracellular amino acids (IC) were isolated from a piece of wet muscle (∼25 mg) by homogenizing on ice using a Teflon pestle with 500 µl of acetonitrile. Samples were centrifuged at 10000×g at 4°C for 5 min and the supernatant containing the IC was collected and dried under nitrogen at 70°C. The remaining mixed protein pellet was washed once with distilled water and 70% ethanol and lyophilized. The dry mixed protein pellets were hydrolyzed with 6 M HCL at 110°C for 24 h. Plasma samples were also analysed for L-[*ring*-^13^C_6_] phenylalanine enrichment to confirm steady-state during the protein synthesis measurement period. Amino acids were converted to their heptafluorobutyric (HFB) derivatives before analysis by GC-MS (models 6890 GC and 5973 MS; Hewlett-Packard, Palo Alto, CA). Plasma and IC phenylalanine enrichments were determined using electron-impact ionization by monitoring ions 316 and 322 (m+0 and m+6, respectively).

Myofibrillar (MYO) and non-myofibrillar (i.e., sarcoplasmic, which includes mitochondrial proteins – ‘SARC’) enriched protein fractions were isolated from a separate piece of wet muscle (∼50 mg), as described above in immunoblotting. The samples were spun at 2200×g for 10 min at 4°C. The resultant supernatants were removed and a small aliquot was taken for immunoblotting and the remaining (∼200 µl) was used to precipitate the sarcoplasmic proteins by the addition of 1 ml of 1 M perchloric acid. The remaining myofibrillar and collagen pellet was washed with 500 µl of distilled water and spun at 700×g for 10 min at 4°C. The myofibrillar proteins were solubilised by adding 1.5 ml of 0.3 M NaOH and heating at 37°C for 30 min with vortex mixing every 10 min. Samples were centrifuged at 10000×g for 5 min at 4°C and the supernatant containing the myofibrillar-enriched fraction was collected and the collagen pellet was discarded. Myofibrillar proteins were precipitated by the addition of 1 ml of 1 M PCA and spinning at 700×g for 10 min at 4°C. The myofibrillar and non-myofibrillar proteins were washed twice with 70% ethanol and the latter was lyophilized. Amino acids were liberated by adding 1.5 ml of 6 M HCL and heating at 110°C for 24 h. Myofibrillar, sarcoplasmic, and mixed muscle proteins were purified using cation exchange chromatography (Dowex 50WX8-200 resin; Sigma-Aldrich) and converted to their N-acetyl-n-propyl ester derivatives for analysis by gas chromatography combustion-isotope ratio mass spectrometry (GC-C-IRMS: Hewlett Packard 6890; IRMS model Delta Plus XP, Thermo Finnigan, Waltham, MA). Derivatized amino acids were separated on a 30m×0.25mm×0.25µm DB-5 column (temperature programme: 110°C for 2 min; 10°C min^−1^ ramp to 240°C; 60°C min^−1^ ramp to 300°C; hold for 5 min) prior to combustion.

### Calculations

The fractional synthetic rates (FSR) of MIX, MYO, and SARC proteins were calculated using the standard precursor-product method:
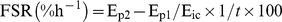
Where, E_p2_ and E_p1_ are the protein bound enrichments from muscle biopsies at time 2 (E_p2_) and plasma proteins or the previous muscle biopsy at time 1 (E_p1_) and thus their difference is the change in bound protein enrichment between two time points; E_ic_ is the mean intracellular phenylalanine enrichment from biopsies at time 2 and time 1; and *t* is the tracer incorporation time. The utilization of “tracer naïve” subjects allowed us to use the pre-infusion blood sample (i.e., mixed plasma protein fraction) as the baseline enrichment (E_p1_) for the calculation of resting MPS. This approach makes the assumption that the ‘natural’ ^13^C enrichment (δ^13^C_PDB_) in the blood reflects that of muscle protein; this is an assumption that has been confirmed in our laboratory [1] and by others [29], [30].

### Statistics

Our mixed design did not permit us to make within subject comparisons therefore, between-condition differences (muscle protein synthesis, anabolic signaling, and gene expression) were tested with a blocked two-factor (condition × time) analysis of variance with repeated measures on time statistics. Acute exercise variables (repetitions, volume load, and time under tension) were analyzed using a one-factor (condition) ANOVA. Linear regression analyses were performed to assess the existence of a linear fit between variables. Pearson's *r* product moment correlation was used to examine the relationship between different variables (muscle protein synthesis and anabolic signaling molecules). Tukey's post hoc test was performed to determine differences between means for all significant main effects and interactions. For all analyses, differences were considered significant at P<0.05. All results are presented as means ± SEM.

## Results

### Acute resistance exercise variables

Acute resistance exercise variables are shown in Table 2. Not surprisingly, the average number of repetitions performed for all 4 sets were significantly different between all conditions (Table 2). Exercise volume (load×repetitions) during exercise was greatest (P<0.05) in 30FAIL condition but was not different (P>0.05) between the 90FAIL and 30WM conditions, which demonstrates we successfully matched external work between the 90FAIL and 30WM exercise modes. Finally, time-under-tension recorded for all 4 sets was significantly different between all conditions (P<0.05).

**Table 2:** Acute unilateral resistance exercise variables during the experimental exercise trial.

	90FAIL	30WM	30FAIL
Load (kg)	82±5* ‡	28±4	28±3
Repetitions^a^	5±0.2	14±0.5†	24±1.1* †
Volume Load (kg)^a^ ^b^	710±30.0	632±28.4	1073±69.9* †
Time under tension (s)^a^	16.3±1.1	27.1±1.85†	43.3±1.9* †

Values are means ± S.E.M.

a Averages for all 4 sets.

b product of repetitions performed multiplied by load (kg) lifted.

*Significantly different from 30WM, P<0.05.

† Significantly different from 90FAIL, P<0.05.

‡ Significantly different from 30WM and 30FAIL, P<0.05.

### Plasma and muscle intracellular phenylalanine enrichments

Muscle intracellular [*ring*-^13^C_6_]phenylalanine (IC) enrichments during trial 1 were 3.0±0.2 tracer·tracee^−1^ at 3 hours after the start of the infusion. Trial 2, IC enrichments were 3.6±0.2, 4.1±0.2, and 4.2±0.3 tracer·tracee^−1^ at 4 hours, for 90FAIL, 30WM, and 30FAIL conditions, respectively; P = 0.27. Trial 3, IC enrichments were 4.1±0.3, 4.8±0.3, and 4.8±0.3 tracer·tracee^−1^ at 3 h for 90FAIL, 30WM, and 30FAIL conditions, respectively; P = 0.50. Plasma [*ring*-^13^C_6_] phenylalanine enrichments are shown in Table 3. Furthermore, linear regression analysis indicated that the slopes of the plasma enrichments were not significantly different from zero (all, P>0.55), suggesting a steady delivery of tracer during the muscle protein synthesis measurements and confirming appropriate conditions for the application of the steady-state precursor product equation.

**Table 3:** Plasma [*ring*-^13^C_6_] phenylalanine enrichments during the experimental trials.

	1 hour	2 hours	3 hours
**Rest**	0.058±0.004	0.059±0.003	0.061±0.002
**Ex**	0.054±0.001	0.062±0.002	0.066±0.003
**PostEx**	0.054±0.003	0.063±0.007	0.060±0.003

Values are means ± S.E.M. Numbers reflect the tracer to tracee ratio (m+6/m+0), see methods for details.

### Muscle protein synthesis

**Figure 1:**
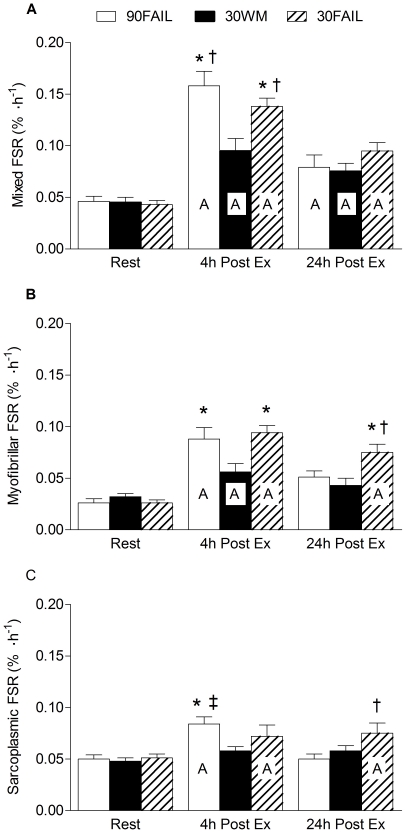
Fasted-state mixed (A), myofibrillar (B), and sarcoplasmic (C) protein synthesis (FSR) at rest and following resistance exercise. Capital letter indicates a mean that is significantly different from rest, P<0.05. *Significantly different from 30WM within that time point, P<0.05. †Significantly different from 90FAIL within that time point, P<0.05. ‡Significantly different from 24h post within group. Values are means ± SEM.

MIX protein synthesis (Fig. 1a) was elevated above rest in all conditions at 4 h post-exercise; however, the increase in MIX protein synthesis above rest at 4 h was greater in the 90FAIL (∼3.5-fold) and 30FAIL (∼3.2-fold) conditions, compared to 30WM (∼2.1-fold) condition. This response was sustained above rest (P<0.05) at 24 h in all conditions with a trend (P = 0.076) towards a greater rate in the 30FAIL than the 30WM condition.

MYO protein synthesis (Fig. 1b) was significantly increased by ∼3.3, ∼3.6, and ∼1.7-fold above rest at 4 h post-exercise in 90FAIL, 30WM, and 30FAIL conditions, respectively; however, the 4 h responses were greater (301% and 279% above rest, respectively both P<0.01) in the 90FAIL and 30FAIL conditions than in 30WM (87% above rest) condition. At 24 h post-exercise, MYO protein synthesis remained elevated (P<0.05) above rest only in the 30FAIL condition (∼2.9 fold). In contrast to the measurements of MIX and MYO protein synthesis, SARC protein synthesis (Fig. 1c) was unchanged in the 30WM condition at 4 h post-exercise, whereas, significant ∼1.7-fold increase was seen in the 90FAIL condition that was also greater than 30WM; however, this rise returned to baseline at 24 h post-exercise. Finally, the 30FAIL condition induced a significant increase in SARC protein synthesis above rest at 4 h (∼1.4-fold) and 24 h (∼1.5-fold greater than rest) post-exercise.

### Anabolic signaling

**Figure 2:**
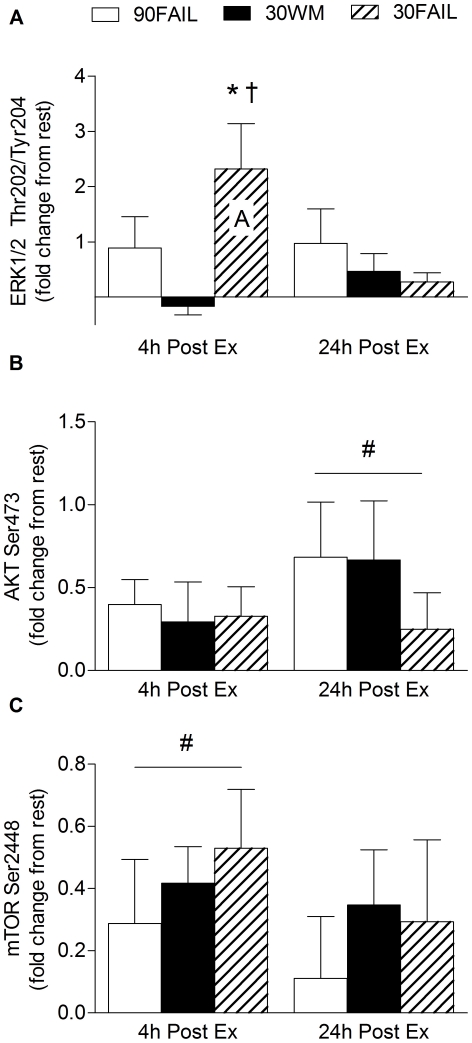
Akt (A), Erk1/2 (B), mTOR (C) following resistance exercise. Capital letter indicates a mean that is significantly different from rest, P<0.05. *Significantly different from 30WM within that time point, P<0.05. †Significantly different from 90FAIL within that time point. #Significant main effect for time, P<0.05. Data are expressed as fold change from rest. Values are means ± SEM.

**Figure 3:**
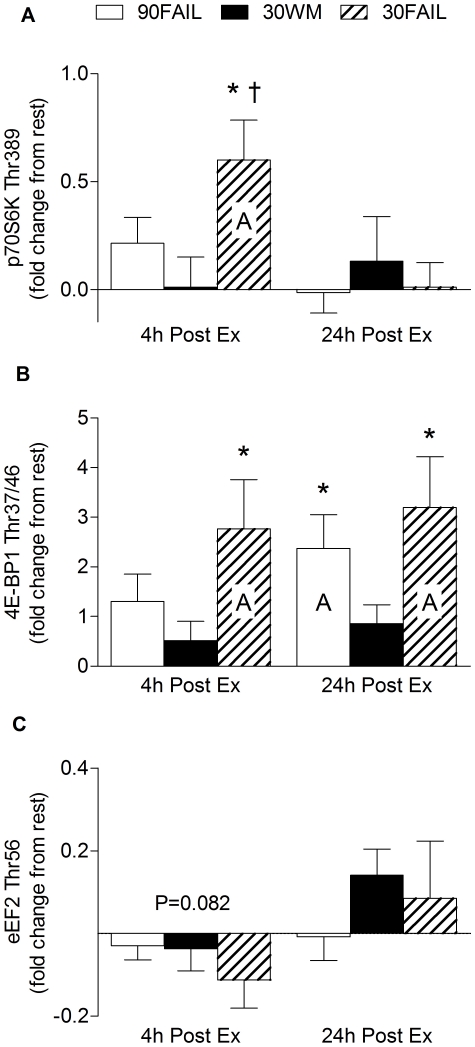
p70S6K (A), 4E-BP1 (B), and eEF2 (C) following resistance exercise. Capital letter indicates a mean that is significantly different from rest, P<0.05. *Significantly different from 30WM within that time point, P<0.05. †Significantly different from 90FAIL within that time point, P<0.05. #Significant main effect for time, P<0.05. Data are expressed as fold change from rest. Values are means ± SEM.

**Figure 4:**
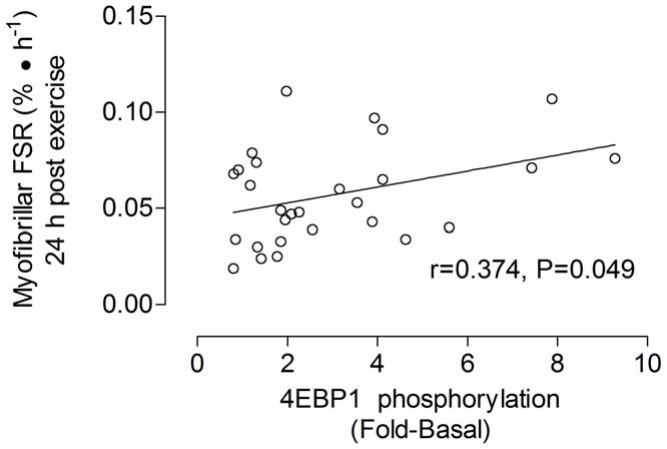
Relationship between myofibrillar protein synthesis and the extent of phosphorylation of 4E-BP1 (Thr 37/46) at 24 hours following resistance exercise. There was a significant correlation (P = 0.049) between the degree of phosphorylation (fold-change from basal) and myofibrillar protein synthesis (FSR, %/hr).

Phosphorylation of Erk1/2^Tyr202/204^ was elevated (P<0.05) at 4 h post-exercise only in the 30FAIL condition (Fig 2a). Akt^Ser473^ phosphorylation was elevated at 24 hours post-exercise regardless of condition (main effect for time, P = 0.023; Fig 2b). Phosphorylation of mTOR^Ser2448^ was elevated to a similar extent at 4 h post-exercise regardless of training condition (main effect for time, P = 0.025; Fig. 2c). As for Erk1/2^Tyr202/204^, phosphorylation of p70S6K^Thr389^ was increased (P<0.05) only in the 30FAIL condition at 4 h post-exercise (Fig. 3a). At 4 h post-exercise, 4E-BP1^Thr37/46^ phosphorylation was only increased in the 30FAIL condition and was sustained at 24 h post-exercise. 4E-BP1^Thr37/46^ phosphorylation became significantly elevated in the 90FAIL condition at 24 h post-exercise (Fig 3b). Linear regression analysis revealed a significant relationship (r^2^ = 0.14, P = 0.049) between the extent of 4E-BP1^Thr37/46^ phosphorylation and the extent of myofibrillar protein synthesis at 24 h post-exercise (Fig 4). The extent of p70S6K activation at 4 h post-exercise was related (although not statistically significant) to the degree of stimulation of myofibrillar protein synthesis at 4 h post-exercise (r^2^ = 0.13, P =  0.055). Finally, eEF2^Thr56^ activation, as indicated by a dephosphorylation, trended to increase above resting levels only in the 30FAIL trial at 4 h post-exercise (Fig 3c).

### Pax7 and MRFs mRNA

**Figure 5:**
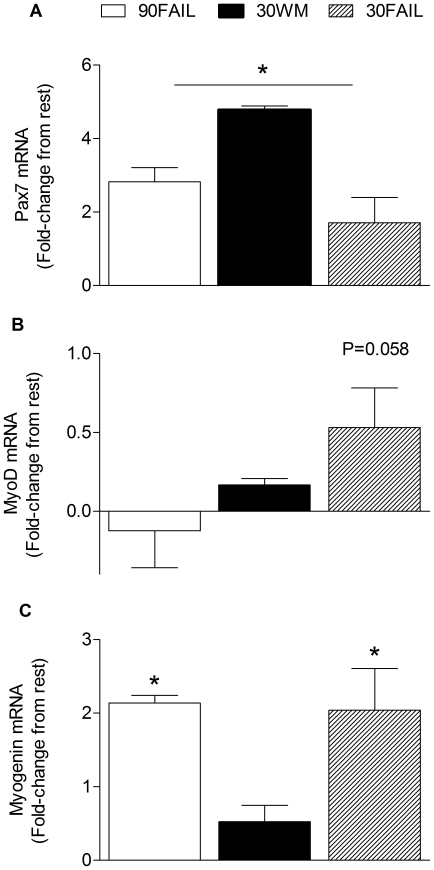
Pax7 (A), MyoD (B), myogenin (C) mRNA expression in skeletal muscle 24 hours following resistance exercise. Data are expressed as fold change from rest. Data are normalized to GAPDH and reported as mean ± SEM. *Significantly different from rest, P<0.05. †Significantly different from 30WM.

Resistance exercise, regardless of load, induced an increase in Pax7 mRNA expression at 24 h post-exercise (main effect for time, P = 0.02; Fig 5a). MyoD mRNA expression trended (P = 0.058) to increase (∼53%) above rest in the 30FAIL condition at 24 h post-exercise (Fig 5b). At 24 h post-exercise, exercise until failure induced a 95% and 204% increase in myogenin mRNA expression in the 90FAIL (P = 0.011) and 30FAIL (P<0.001) conditions, respectively (Fig 5c). Furthermore, myogenin mRNA expression was greater (P = 0.026) in the 30FAIL condition as compared to that in the 30WM condition at 24 h post-exercise (Fig 5c). There were no changes in MRF4 or Myf5 mRNA expression from rest (data not shown).

## Discussion

We report for the first time that low-load high volume resistance exercise (30FAIL) is more effective at increasing muscle protein synthesis than high-load low volume resistance exercise (90FAIL). Specifically, the 30FAIL protocol induced similar increases in MYO protein synthesis to that induced by the 90FAIL protocol at 4 h post-exercise but this response was sustained at 24 h only in 30FAIL. Furthermore, the MYO response in 30WM are in agreement with those of Kumar and colleagues [6] who demonstrated a dose-dependent relationship of exercise-load when volume (i.e., external work—expressed as repetitions×load) is equalized. In contrast to recommendations [31], that heavy loads (i.e., high intensity) are necessary to optimally stimulate MYO protein synthesis, it is now apparent that the extent of MYO protein synthesis after resistance exercise is not entirely load dependent, but appears to be related to exercise volume and, we speculate, to muscle fibre activation and most likely to the extent of type II fibre recruitment.

These results do agree with previous suggestions [31], [32], that the duration of the MYO response may be determined by exercise volume at extended time points beyond the exercise bout. It is worth highlighting, however, the early amplitude of MYO protein synthesis is dependent on contraction intensity as indicated by a greater response of muscle protein synthesis in the 90FAIL and 30WM conditions (Fig. 1b). This suggests that the volume of exercise, which we view as being related to the degree of fibre activation affects the duration and intensity affects the acute amplitude of the MYO response.

We found that resistance exercise was stimulatory, albeit to a lesser extent than that seen for MIX or MYO protein synthesis, on SARC protein synthesis at 4 h post-exercise for the 90FAIL (∼1.7-fold increase above rest) and 30FAIL (∼1.4-fold increase above rest) conditions but similar to the MYO results, the response was only sustained for 24 h in the 30FAIL condition. This finding is somewhat contradictory to our previous findings demonstrating that resistance exercise did not offer any further benefit on the SARC protein fraction when feeding a 25 g bolus of protein alone [24]. Indeed, others have shown a stimulatory effect on fed-state SARC protein synthesis utilizing a greater volume of resistance exercise [33] than we used [24] or with a more aerobic-based exercise stimulus [17], [32]. Regardless, comparisons between the studies are difficult to reconcile as different feeding (bolus vs. pulsed feeding patterns) and exercise modalities were utilized. However, the results of the current study demonstrate that resistance exercise, under physiological conditions, can stimulate fasted-state SARC protein synthesis and exercise volume may be of considerable importance in sustaining this response. It should be noted that the 30FAIL condition induced the greatest sustained (elevated at both 4 h and 24 h) rise in SARC (which includes mitochondria proteins). We propose that this finding provides support for the idea that 30FAIL exercise mode may function as an exercise mode to increase proteins from all fractions in muscle including mitochondrial and myofibrillar proteins leading to both enhanced oxidative capacity and hypertrophy. Such a proposal would of course require testing in a chronic situation.

It is generally accepted that the MIX protein synthetic response serves as an appropriate surrogate marker for MYO protein synthesis [34], as a result of the predominance of MYO proteins in muscle (∼60–65% of total MIX protein). However, our laboratory has demonstrated the importance of examining muscle protein specific sub-fractions on several occasions [15], [24], [35] and we present here another example of this differential response between MIX and MYO. Specifically, there were greater elevations in MYO and SARC in 30FAIL that were not seen in MIX. Also, a divergent 24 h post-exercise responses in 90FAIL and 30WM conditions, as noted by sustained responses in MIX protein synthesis, whereas MYO protein synthesis returned to baseline values in these two exercise conditions. The reason for sustained increase in MIX protein synthesis in the 90FAIL and 30WM conditions may be manifested in the SARC fraction which represents ∼30% of MIX protein and has been established to turn over at a rate that is twice that of myofibrillar proteins [24], [36], [37]. This notion is further supported by the current data (Fig. 1c). In addition, muscle collagen, which composes about ∼10–15% of mixed muscle protein, has been established as exhibiting markedly elevated rates after exercise [32], [38] and thus could be contributing to the sustained response at 24 h in MIX protein synthesis observed in the current study. These points are not trivial since studies examining MIX protein synthesis have provided the framework on which much of our current understanding of how the muscle responds to different exercise and feeding interventions occurs. Our data clearly indicate a need to examine in greater detail, including protein sub-fraction measurement, rates of protein synthesis.

A surprising finding was that 24 h after the resistance exercise bout, regardless of exercise load, Akt phosphorylation was elevated (main effect for time). This result was seen even though the subjects reported to the lab in the fasted state, which is confirmed by plasma insulin levels at 5.1±1.2 µU/ml at baseline. It has previously been reported that Akt is exceptionally responsive to nutrients [39] and physiologically high plasma insulin [40]. Indeed, many investigations have established that Akt is transiently phosphorylated following exercise [14], [41], [42], [43] or even no change [12], [44], [45]. To our knowledge only one study has examined the phosphorylated-state of Akt at 24 h following acute resistance exercise [46]. Although our findings are contrasting, it is difficult to reconcile the physiological significance of the elevated phosphorylation of a signalling protein (i.e., Akt) that is so distal within the signalling cascade, especially since a non-linear relationship between anabolic signalling molecules and muscle protein synthesis has been established to exist [40]. However, it is important to recognize that latent increases in anabolic signalling proteins the following day after exercise is not entirely novel [23].

The phosphorylated-state of mTOR was also elevated in all three exercise conditions at 4 h post-exercise; however, one of its downstream targets p70S6K was only phosphorylated in the 30FAIL condition. Similarly, Erk1/2 was significantly activated only in the 30FAIL condition at 4 h post-exercise, which provides further evidence that tandem activation of p70S6K with mTOR is occurs in human muscle [47], as previously suggested in other model systems [46], [48].

It has been suggested that Erk1/2 is sensitive to the number of contractions performed during the exercise bout [49], which is supported by our results because ∼94 repetitions in total performed in the 30FAIL condition stimulated greater Erk1/2 activation than ∼19 and ∼62 repetitions performed in the 90FAIL and 30WM conditions, respectively.

In addition, we have demonstrated that 4E-BP1 is activated at both 4 h and 24 h post-exercise in the 30FAIL condition; however, a latent increase in 4E-BP1 phosphorylation was observed at 24 h post-exercise in the 90FAIL condition. To our knowledge, the relationship between the extent of 4E-BP1 phosphorylation and the extent of elevation of myofibrillar protein synthesis at 24 h post-resistance exercise has never been reported (Fig 3). Certainly, with the abundance of anabolic signalling data obtained in close temporal proximity to the exercise bout and their activation decreasing beyond the end of exercise [6], [12] suggests that other mechanisms are in place to maintain synthetic rates in the hours and possibly days after exercise [19]. Whereas these mechanisms remain relatively undefined, it is possible that 4E-BP1 may, in part, be having some controlling effect. Finally, our results provide some confirmation that the idea that p70S6K and 4E-BP1 are important proximal regulators muscle protein synthesis. This notion is based on observations from current study and other data demonstrating that p70S6K phosporylation is correlated with rates of MYO protein synthesis after an acute bout of resistance exercise in young men [6] and that the degree of phosphorylation of p70S6k and 4E-BP1 may be related to the exercise load and/or the number of contractions performed [6].

It has previously been suggested that resistance exercise load has no influence on MRFs mRNA expression (Myo-D, myogenin, MRF-4, and myf5) at 0.5, 1, or 2 h post-exercise [50], but in that study there was no attempt to equate volume between low-load or high-load exercise bouts. Moreover, the use of similar exercise intensities, ∼65% 1RM and ∼85% 1RM likely precluded the researchers from detecting differences [50]. Clearly, to determine if differences do exist in MRFs gene expression in response to different loads, then equating exercise volume (i.e., external work) is paramount. In the current study we found the expression of MyoD mRNA, which is associated with satellite cell commitment to myogenic lineage and the resultant proliferation of the myoblast [22], trended (204%, P = 0.058) to be greater than the value at rest at 24 h post-exercise in the 30FAIL condition; whereas, mRNA for myogenin mRNA, which mediates terminal differentiation of the myoblast [22], was up-regulated by ∼2 and 3-fold respectively in the 90FAIL and 30FAIL conditions. A novel finding of the current work was the divergent response in myogenin mRNA expression between the 30WM and 30FAIL conditions. Specifically, these results suggest that the extent of muscle fiber activation (i.e., the extent along the pathway until failure during exercise) had an impact on the extent of myogenin expression at 24 h after resistance exercise. This thesis is further supported by the myogenin response in the 90FAIL condition in that it was also significantly elevated above rest, which is in contrast to the result in the low load work-matched (i.e., 30WM) condition. Collectively, our data suggest that myogenin mRNA expression is unrelated to intensity; however, exercise volume, which we interpret as a proxy for the extent of muscle fibre activation or disruption of cellular homeostasis, likely to be driving the response.

We are unable to discern whether the up-regulated MyoD or myogenin mRNA expression occurred in satellite cells, postmitotic myonuclei, or was due to the stabilization of existing mRNA, however, the fact that at 24 h after exercise Pax7 mRNA expression was up-regulated, regardless of load, suggests that satellite cells were activated as Pax7 expression is specific to satellite cells [21]. Indeed, we demonstrated a robust Pax7 mRNA response above rest in the 30WM condition (∼5.7-fold) at a time when MYO protein synthesis had returned to basal. This is not entirely surprising, as it is unlikely that the satellite cell response drives the muscle protein synthetic response and thus, we can likely separate the acute muscle protein synthetic response from that of the acute satellite cell response. This notion is supported by the fact that MRFs expression can be detected ≥120 h post-exercise recovery [27], a time point beyond which increases in muscle protein synthesis after resistance exercise are seen [18], [19].

Based on assumption that acute changes in muscle protein synthesis are predictive of phenotypic adaptations, our data would suggest that a high volume low-load resistance exercise paradigm may serve as an excellent training paradigm to attenuate age-related sarcopenia, and other frailty and wasting conditions by maintaining and/or inducing skeletal mass while at the same time minimizing the potential for both orthopaedic and soft tissue injury in these susceptible populations. This notion is supported by data which demonstrated that aged muscle (∼70 years) has an ‘anabolic resistance’ to resistance exercise [6] and this resistance may be overcome with increased volume of exercise [51]. Finally, while it appears that low load paradigms utilizing blood flow restriction during exercise, induced via a pressure cuff are effective at inducing an anabolic response in both young [7] and older subjects [47], the low-load high volume training employed in the current study may provide a more practical alternative for inducing hypertrophy and/or attenuating sarcopenia. However, it is important to recognize that acute scientific studies simply supply the framework on which to build future training studies upon to directly test if a cause-and-effect relationship does in fact exist.

In conclusion, we have demonstrated that low-load high volume resistance exercise has a potent stimulatory effect on anabolic signalling molecules, MyoD and myogenin mRNA expression and muscle protein synthesis. Our results support previous findings that demonstrated after 16 weeks of isometric training at 30% maximal voluntary contraction that significant increases in fibre area can be achieved [52]. Although, the contraction type employed in the current study (i.e., dynamic) differed from Alway and colleagues [52] (i.e., isometic), our data provides further support that low-load contractions performed with numerous repetitions or high-load contractions performed for fewer repetitions will result in similar training induced gains in muscle hypertrophy as previously suggested [52], or even superior gains, as results from the current study would predict. This premise is further supported by data which demonstrates that short-term changes in muscle protein synthesis [1], [2] are predictive of training induced gains in muscle mass [3], [4]; however, a training study in which these distinctly different exercise loads (90FAIL and 30FAIL) are utilized is clearly warranted to confirm our speculation.
